# Prevalence of five treatable sexually transmitted infections among women in Lower River region of The Gambia

**DOI:** 10.1186/s12879-023-08399-2

**Published:** 2023-07-13

**Authors:** Robert Butcher, Sheikh Jarju, Dolapo Obayemi, Adedapo Olufemi Bashorun, Hristina Vasileva, Hannah Bransbury-Hare, Orighomisan Agboghoroma, Lamin Drammeh, Martin Holland, Emma Harding-Esch, Ed Clarke

**Affiliations:** 1 Clinical Research Department, London School of Hygiene & Tropical Medicine, London, UK; 2 MRC Unit The Gambia at London School of Hygiene & Tropical Medicine, London, UK

**Keywords:** Sexually transmitted infections, Gambia, Prevalence, *Chlamydia trachomatis*, *Trichomonas vaginalis*, Syphilis, *Mycoplasma genitalium*, *Neisseria gonorrhoeae*

## Abstract

**Background:**

The prevalence of sexually transmitted infections (STIs) in sub-Saharan Africa is poorly described. We aimed to determine the prevalence of five treatable STIs (*Chlamydia trachomatis, Neisseria gonorrhoeae, Trichomonas vaginalis, Mycoplasma genitalium, Treponema pallidum*) in a sample of Gambian women from the general population.

**Methods:**

Archived specimens from 420 women aged 15 − 69 years living in The Gambia enrolled in a clinical trial of human papilloma virus vaccine schedules were tested in this study. Urine samples were tested for *C. trachomatis*, *N. gonorrhoeae*, *T. vaginalis* and *M. genitalium* using a commercially available, open-platform multiplex PCR kit. A fragment of the *ompA* gene was amplified from *C. trachomatis*-positive samples and sequenced. Serum samples were tested for *T. pallidum* using the Chembio DPP Syphilis Screen and Confirm test.

**Results:**

Overall, 41/420 (9.8%) women tested positive for at least one STI. 32 (7.6%), 9 (2.1%), 1 (0.2%), 1 (0.2%) and 0 (0.0%) tested positive for *T. vaginalis*, *C. trachomatis*, *N gonorrhoeae*, *M. genitalium* and *T. pallidum*, respectively. *ompA* gene sequence was available from five *C. trachomatis* infections: four were genovar D,one was genovar G and one was genovar F.

**Conclusions:**

STIs are endemic in The Gambia. Monitoring systems should be established.

**Supplementary Information:**

The online version contains supplementary material available at 10.1186/s12879-023-08399-2.

## Background

The World Health Organization (WHO) estimates that there are one million new cases of sexually transmitted infections (STIs) each day globally caused by a range of bacterial, viral and protozoan pathogens [1]. STIs can significantly impact reproductive health, can cause adverse outcomes during pregnancy and childbirth, and can be vertically transmitted to a new born [2]. Despite ongoing efforts to control transmission, the prevalence and incidence of many common STIs is thought to be stable or, for example in the case of *Chlamydia trachomatis*, increasing in several countries [3, 4]. Regional data synthesised from Africa suggest the prevalence of key curable STIs is particularly high and increasing [1]. However, available data come from a limited number of high-income countries, with data from low- and middle-income countries either lacking or coming from targeted or high-risk subgroups [1, 5]. The heterogeneity in data availability, combined with the variability in sampling and data collection methods can lead to uncertainty in disease rates which, in itself, can impair control efforts [6–8]. Case recording and management are often syndromic in resource-limited settings as molecular diagnostics are expensive, require specialist infrastructure and are hence unavailable [9]. However, since the majority of STI cases are asymptomatic, the lack of routine diagnostic testing is another barrier to understanding the global burden of infections [10]. There is an urgent need to generate more data on the epidemiology of STIs in low- and middle-income countries, first, to determine where interventions are needed and second, to provide baseline estimates against which future intervention efforts can be evaluated. STI prevalence is also known to vary widely within and between communities due to many sociocultural, behavioural and host − pathogen interactive factors. It is, therefore, critical to represent diverse populations in global STI surveillance efforts.

Studies in the early 1980s from antenatal clinics in Bakau, The Gambia, suggested that almost 50% of women attending had *Neisseria gonorrhoeae*, *C. trachomatis*, *Trichomonas vaginalis* or *Treponema pallidum* [11, 12]. In 2017, 14% of women attending antenatal clinics near Banjul, The Gambia, tested positive for *N. gonorrhoeae*, *C. trachomatis*, *T. vaginalis* or *T. pallidum* [13]. In rural communities in the Western Division of The Gambia in 1998, 0.5% and 4.4% of survey participants tested positive for *C. trachomatis* and *T. pallidum*, respectively [14]. Data on the prevalence of STIs among general populations are still uncommon, and no previous studies have been conducted using molecular techniques (the gold standard in terms of diagnostic accuracy) for *C. trachomatis*, *N. gonorrhoeae*, *M. genitalium* and *T. vaginalis* [15]. In this study, we used nucleic acid amplification tests for four prevalent treatable STIs (*C. trachomatis*, *N. gonorrhoeae*, *T. vaginalis*, *Mycoplasma genitalium*) and serological tests for *T. pallidum* to determine their prevalence in Gambian women from the general rural population.

## Methods

### Study setting and samples

The study used anonymised, archived urine and serum samples collected as part of an ongoing clinical trial of human papilloma virus (HPV) vaccine dosing schedules in The Gambia (the HANDS trial; https://clinicaltrials.gov/ct2/show/NCT03832049; date registered: 06/02/2019; clinicaltrials.gov registration reference: NCT03832049). The participants were recruited from Jarra Soma, a transit point on the trans-Gambian highway in the Lower River Region, and a further eight surrounding rural villages. Through community and school sensitisation, communities and school childrens’ guardians were made aware of the study, allowing interested potential participants to approach the study team. Eligible participants for the trial were females aged 4–26 years who lived in the study area. Potential participants were excluded if they had a significant chronic illness, known or suspected human immunodeficiency virus (HIV) infection, and if they were pregnant or planning to become pregnant in the six months of study participation. Participants were asked to complete a questionnaire to report their age, ethnic group, primary cooking fuel, water source, sanitation, education level, age at menarche, marriage status, occupation, and contraceptive use. Questionnaires included questions with categorial answers. Local knowledge was used to generate the categorical list of potential responses.

At the time of enrolment to the parent trial, before any trial interventions had taken place, urine and serum were collected from participants (≥ 15 years) or their mothers/female guardians (for participants < 15 years) for HPV genotypic and seroprevalence testing. The samples used in this study came from those who provided additional informed consent for the future use of retained samples for other ethically approved research. Specimens were collected between September 2019 and February 2021 and tested for the STI prevalence study in May 2021.

### Specimen collection and processing

First-void urine (FVU) was self-collected directly into nucleic acid preservative using the Colli-Pee™ device (Novosanis, Antwerp, Belgium), [16] prior to aliquoting and storage at -80 °C before further processing. Blood samples for serum separation were collected by peripheral venepuncture into Serum Separation Tube™ (BD Biosciences, Wokingham, UK). Having been allowed to clot for at least 30 min at ambient temperature, serum was separated by centrifugation, aliquoted and stored at -80 °C before further processing.

### Pathogen detection

DNA was extracted from 100 µL of FVU using the AmpliSens DNA-sorb-AM extraction kit (InterLabService Ltd, Moscow, Russia) and eluted into 100 $\mu$L of tris ethylenediaminetetraacetic acid (EDTA), according to manufacturer’s instructions. Presence of *C. trachomatis*, *N. gonorrhoeae*, *T. vaginalis* and *M. genitalium* was tested using a commercially available multiplex quantitative PCR assay (AmpliSens kit, multiprime-FRT variant [InterLabService, Moscow, Russia]) run on a BioRad CFX96 (Biorad, Hercules, CA, USA) platform, according to the manufacturer’s protocol [17]. Each sample was tested in a single well containing the reaction mix and 10 $\mu$L of purified DNA. Manufacturer-provided positive exogenous control material (internal control) was added to each well, and manufacturer-provided positive and negative control samples were run on each plate. FVU sample test results were considered valid if they were run on a plate where the assay positive controls amplified, where no amplification was detected in negative controls and where the internal control amplified. Positive and negative results for *C. trachomatis*, *N. gonorrhoeae*, *M. genitalium* and *T. vaginalis* were called according to manufacturer’s recommendations [17].

Serum samples were tested for *T. pallidum* using the Dual Path Platform (DPP) Syphilis Screen and Confirm kit test (Chembio, Hauppauge, NY, USA), [18, 19] according to manufacturer’s instructions. Serum tests for *T. pallidum* were considered valid if the control line was visible. An individual was considered to have a current syphilis infection if both treponemal (indicative of seropositivity for *T. pallidum*, equivalent to a positive *T. pallidum* particle agglutination test result) and non-treponemal (indicative of IgG or IgM anti-cardiolipin antibodies, equivalent to a high-titre Rapid Plasma Reagin test result) lines were visible, and a past syphilis infection if only the treponemal line was visible.

### Chlamydia trachomatis ***ompA*** sequencing

The serotype of the *C. trachomatis* infections identified was determined by sequencing of the *ompA* gene and comparison to published sequences. A 972-bp fragment of *ompA* was amplified using a previously described nested PCR reaction [20]. Products were normalised to 10 ng/$\mu$L, subjected to PCR using the BigDye Terminator v3.1 Cycle Kit (Applied Biosystems, Waltham, MA, USA) and sequenced using a 3730 DNA Analyser (Applied Biosystems, Walthm, MA, USA) by Source BioScience (Cambridge, UK).

### Data analysis

Proportions and confidence intervals (calculated with the Wilson method using the Hmisc::binconf function in R [21, 22]) were presented in crude form and not adjusted or post-stratified. Association between STI status, demographic and behavioural data were tested using logistic regression using the glm(family=”binomial”) function in R [22]. All variables were tested in univariable analyses. There was no evidence of an association between any variable and having any STI in univariable analyses, so no multivariable analyses were performed.

Raw sequence data were imported into R for analysis using the SangerSeqR package [23]. Read ends with a mean phred score < 20 across a 10-base sliding window were trimmed. Where the primary:secondary peak ratio was ≤ 0.33, the primary base was called, otherwise an N was assigned. The forward and reverse-complement of reverse reads were aligned using MUSCLE software to create consensus sequences. The consensus sequences were formed based on three rules: [1] where the base in both reads match, the base is retained; [2] where there are different bases in both reads, an N is assigned in the consensus sequence; [3] where there is a gap in one read and a base is called in the other, the base is called in the consensus sequence. Sequences were queried using the Basic Local Alignment Search Tool (BLAST) using a standard nucleotide query (BLASTN) against NCBI nucleotide databases and the serovar of the best reference strain match was assigned to the sample.

### Study ethics

Both the HANDS trial (SCC1597) and the STI prevalence study (21700) were approved by the MRC Gambia Scientific Coordinating Committee, The Gambia Government/MRC Joint Ethics Committee and the London School of Hygiene & Tropical Medicine Observational Ethics Committee. The study adhered to the tenets of the Declaration of Helsinki.

## Results

### Participant overview

FVU, serum, and matched clinical data were available from 420 women. This included 212 15- to 26-year-old HANDS trial participants and 208 mothers/female guardians of younger participants. Of the available archived HANDS trial specimens from 427 women, there were five urine samples without paired serum samples, one urine sample where the internal control did not amplify and one serum DPP test where a control line was not visible so was considered invalid. These seven specimens were excluded from the analysis.

The median age of the study population was 25 years (range: 15 − 69 years; interquartile range: 19 − 36 years). The median age at menarche of the 375 study participants who could recall was 15 years (range: 10 − 20 years; interquartile range: 14 − 15 years). The majority (86%) of the women tested were of Mandinka ethnicity. One-third (33%) had never attended school. In total, 59% were married and 30% were currently using or had previously used contraception, predominantly injectables. A summary of the study population ethnicity, education status, marital status and contraception-use is shown in Table 1.

**Table 1:** Descriptive characteristics of 420 women from The Gambia tested for five treatable sexually transmitted infections

Variable	Level	Count (n = 420) (%)
Ethnicity	Mandinka	362 (86)
	Fula	36 (9)
	Other (Jola, Serahule, Serere, Wolof, other)	22 (5)
Education level	None or can’t remember	157 (37)
	Lower Basic (grades 1 − 6)	50 (12)
	Upper Basic (grades 7 − 9)	103 (25)
	Senior Secondary (grades 10 − 12)	101 (24)
	Diploma/equivalent	9 (2)
Marital Status	Married	248 (59)
	Single	161 (38)
	Separated, divorced or widowed	11 (3)
Past or current contraceptive use	No	293 (70)
	Yes*	127 (30)
	Injectable contraceptive	106
	Oral contraceptive	26
	Condom or other barrier method	2
	Intrauterine device	2

* Some participants reported using more than one contraceptive method

### Prevalence of sexually transmitted infections

Of the 420 women included, 41 (9.8%) tested positive for at least one STI. *T. vaginalis* was the most prevalent (7.6%), while there were no cases of *T. pallidum* infection. The proportion of tested women with each STI is shown in Table 2.

There were three women (0.7%) who had a visible non-treponemal line and four (1.0%) who had a visible treponemal line on DPP testing. None had both lines.

**Table 2:** Proportion of 420 women from The Gambia with a positive sexually transmitted infection test result, presented by causative agent

Infection	Negative	Positive	% (95% confidence interval)
*Chlamydia trachomatis*	411	9	2.1 (0.9 − 4.0)
*Neisseria gonorrhoeae*	419	1	0.2 (0.0 − 1.3)
*Trichomonas vaginalis*	388	32	7.6 (5.3 − 10.6)
*Mycoplasma genitalium*	419	1	0.2 (0.0 − 1.3)
*Treponema pallidum*	420	0	0.0 (0.0 − 0.9)
**Any infection**	**379**	**41**	**9.8 (7.1 − 13.0)**
One infection		39	-
More than one infection		2	-

**Fig. 1:**
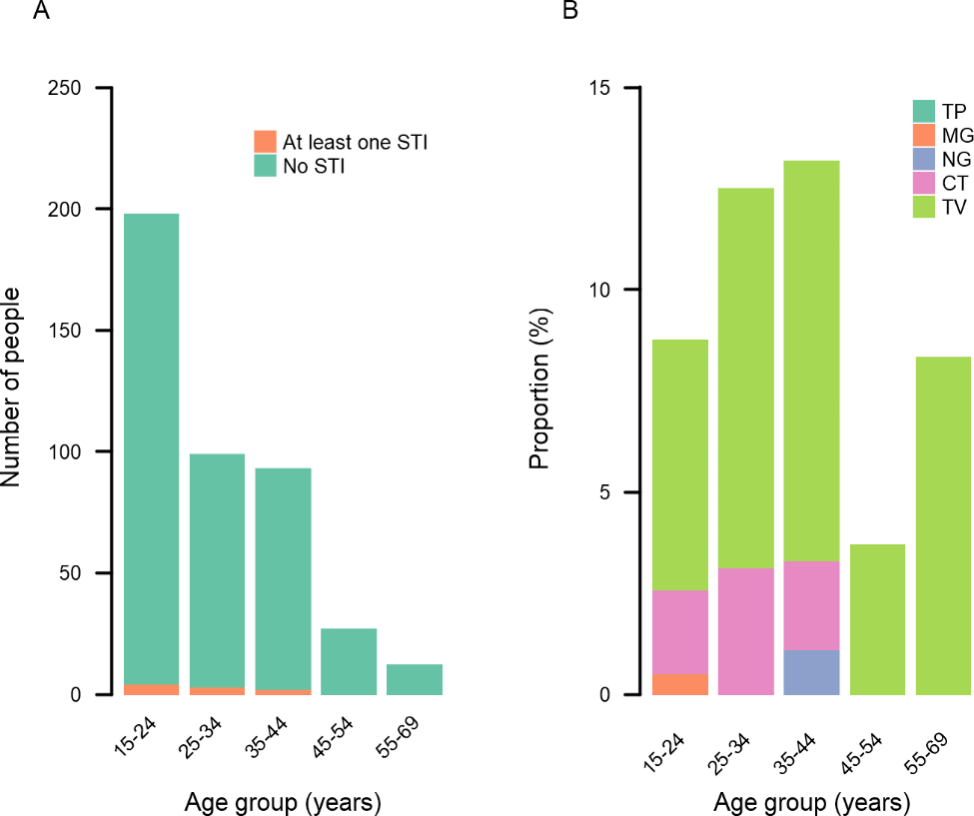
**A.** Number women from The Gambia testing negative and positive for at least one sexually transmitted infection, presented by age group. **B.** Proportion of women from The Gambia testing positive for syphilis (TP), *Mycoplasma genitalium* (MG), *Neisseria gonorrhoeae* (NG), *Chlamydia trachomatis* (CT) and *Trichomonas vaginalis* (TV), presented by age group

STIs were most common in the 35 − 44-year-old age group, of whom 13% tested positive for at least one STI (Fig. 1A; Table 3). *T. vaginalis* was detected in participants of all age groups, whereas *M. genitalium* was only present in those aged 15–24 years, *N. gonorrhoeae* only in those aged 35–44, and *C. trachomatis* in those aged under 45 years (Fig. 1B; Table 3). There was no evidence of an association between any variable and STIs in univariable analysis (supplementary Table 1).

**Table 3:** Age-specific prevalence of sexually transmitted infections in 420 women from The Gambia, presented in ten-year age groups

Age group (years)	*N*	*T. vaginalis* % (95% CI)	*C. trachomatis* % (95% CI)	*N. gonorrhoeae* % (95% CI)	*M. genitalium* % (95% CI)	*T. pallidum* % (95% CI)	Any infection % (95% CI)
15 − 24	194	6.2 (2.8 − 9.6)	2.1 (0.1 − 4.1)	0.0 (0.0 − 1.9)	0.5 (0.0 − 2.8)	0.0 (0.0 − 1.9)	8.2 (4.8 − 13.0)
25 − 34	96	9.4 (3.6 − 15.2)	3.1 (0.0 − 6.6)	0.0 (0.0 − 3.8)	0.0 (0.0 − 3.8)	0.0 (0.0 − 3.8)	11.5 (5.9 − 19.6)
35 − 44	91	9.9 (3.8 − 16.0)	2.2 (0.0 − 5.2)	1.1 (0.0 − 6.0)	0.0 (0.0 − 4.1)	0.0 (0.0 − 4.1)	13.2 (7.0 − 21.9)
45 − 54	27	3.7 (0.0 − 10.8)	0.0 (0.0 − 12.5)	0.0 (0.0 − 12.5)	0.0 (0.0 − 12.5)	0.0 (0.0 − 12.5)	3.7 (0.0 − 19.0)
55 − 69	12	8.3 (0.0 − 23.9)	0.0 (0.0 − 24.2)	0.0 (0.0 − 24.2)	0.0 (0.0 − 24.2)	0.0 (0.0 − 24.2)	8.3 (0.2 − 38.4)

*ompA* gene fragments were amplified from six of the nine *C. trachomatis*-positive DNA extracts. The mean sequence length was 936 bases (range: 929 − 943). Four of these aligned most closely to published serovar D sequences, one aligned most closely to published serovar G sequences and one aligned most closely to serovar F sequences.

## Discussion

We found approximately 10% of women in Lower River Region of The Gambia testing positive for at least one STI, with the highest prevalence being for *T. vaginalis* whilst no *T. pallidum* was detected.

The proportion of women testing positive for an STI was comparable to findings from other STI prevalence studies from the region. A meta-analysis of data from several countries estimated the WHO Africa region (AFRO) prevalence of *C. trachomatis*, *N. gonorrhoeae*, *T. vaginalis* and *T. pallidum* to be 5.0 (3.8–6.6), 1.9 (1.3–2.7), 11.7 (8.6–15.6) and 1.6% (1.2–2.0%), respectively, in 2016 [1]. Our findings are 2.1 (0.9 − 4.0), 0.2 (0.0 − 1.3), 7.6 (5.3 − 10.6) and 0% (0.0 − 0.9%), respectively, thus following a similar trend in relative prevalence. The WHO AFRO covers a large and diverse region, and the population of The Gambia only makes up a small proportion of the regional population, so comparison to these regional estimates should be considered accordingly.

Despite the variation in sampling and testing methods used in other studies, the similarity between these findings and previous studies was notable. At almost 8%, our data suggest *T. vaginalis* is the most prevalent STI in this population. Other studies in West Africa estimate the prevalence of *T. vaginalis* to range from 2 − 6%, although several used wet mount microscopy rather than molecular tests, [24–27] so the estimates are not directly comparable [28]. *T. vaginalis* is often found to be highly prevalent, particularly in Africa [1]. At < 1%, the proportion of women with *N. gonorrhoeae* in this population from The Gambia was low, and lower than several similar studies from the region, where estimates ranged from 1 − 4%, [26, 27, 29, 30] including a recent study from an urban coastal region in The Gambia where the prevalence of *N. gonorrhoeae* was 2% [13]. We found an *M. genitalium* prevalence of 0.2%. This is lower than expected, as from the limited population-based studies reported elsewhere, prevalence is estimated to be 3.9% across low-income settings [31]. The proportion of *C. trachomatis*-infected women in this study was 2.1% (0.9 − 4.0%). Although other estimates of *C. trachomatis* prevalence from the region show considerable heterogeneity, this is generally in keeping with prevalence estimates from neighbouring countries [26, 27, 29, 30]. To find no women with active *T. pallidum* infection was surprising given other estimates of *T. pallidum* prevalence from elsewhere in The Gambia. It is possible that the three positive non-treponemal test results were active cases of *T. pallidum*. However, because of the well-described risk of false positive non-treponemal test results from other non-treponemal infections, such as malaria, [32] in the absence of a positive treponemal test result we cannot say definitively that these were caused by *T. pallidum*. This diagnostic algorithm for *T. pallidum* is in keeping with recent guidelines from other organizations [33]. The four positive treponemal test results in the absence of positive non-treponemal test results suggests some historic exposure to *T. pallidum* but not active infection. Together, these data suggest some ongoing *T. pallidum* transmission in the population, albeit likely at a low level given the general low prevalence relative to estimates found elsewhere.

WHO identified strengthening STI surveillance and improving knowledge of prevalence (alongside symptom aetiology and antimicrobial resistance) as priorities in their STI strategy for 2016–2021 [34]. Sexual and reproductive health services in The Gambia should be strengthened to manage this burden of STIs. Controlling the prevalence of *T. vaginalis* should be a particular focus. Having *T. vaginalis* may increase risk of HIV acquisition [35] and may have adverse impact on pregnancy outcomes, [36] although the evidence is inconsistent [37]. Only 5% of *T. vaginalis* infections currently exhibit some resistance to first-line therapy, [38, 39] but there remain concerns of antimicrobial resistance (AMR) emerging [40] *C. trachomatis* is a pathogen which is generally thought to be increasing in some countries, [3, 4] so the low prevalence found here is notable. We found serovars D, G and F in this sample set, which were the three most common *C. trachomatis* serovars in a 2012 study from nearby Guinea Bissau [41]. The low *N. gonorrhoeae* prevalence is reassuring, given *N. gonorrhoeae*’s propensity to develop extensive drug resistance[42, 43] resulting in it being on WHO’s “high priority” list for new antibiotics [44]. *M. genitalium* is another pathogen where AMR is of increasing concern [43, 44]. However, in a systematic review of mutations associated with macrolide and fluoroquinolone resistance in *M. genitalium*, AMR data for the African region were only available from Kenya and South Africa [45]. Integrating AMR surveillance with prevalence and incidence monitoring would be valuable in this setting, particularly considering the amount of community-wide exposure to antimicrobials imparted by neglected disease mass drug administration campaigns in The Gambia and its neighbouring countries [46]. As part of this work, we attempted to amplify AMR-associated single nucleotide polymorphism-containing regions from the residual ex-diagnostic DNA eluate of positive samples. We found a product to amplify in < 50% samples and generated several low-quality sequences among the gene segments which did amplify. Therefore, more reproducible methods should be employed for future AMR surveillance.

The study has two key strengths. First, data in this study were collected from a sample of the general population in sub-Saharan Africa. General population samples are more generalizable to the wider population than some other common STI survey sampling methods, such as sampling of clinic attendees or high-risk groups. No published evidence of STI prevalence in this population were available at the time of the study, and very few data on STIs were available from The Gambia as a whole. Also, low- and middle-income countries in sub-Saharan Africa are under-represented in global estimates of STI burden. Second, recommended diagnostic approaches were used to detect STIs in this study: nucleic acid amplification tests for diagnosis of *C. trachomatis*, *N. gonorrhoeae*, *M. genitalium* and *T. vaginalis* and a specific serological diagnostic algorithm for detection of *T. pallidum*. These methods are essential for detecting asymptomatic carriage of STIs and offer improved sensitivity over culture or antigen detection methods.

There were also limitations to this study. First, for detection of *C. trachomatis*, *N. gonorrhoeae*, *T. vaginalis* and *M. genitalium*, we tested FVU. Despite its convenience as a sampling method, FVU is recognised as a difficult sample type to amplify DNA from and a less sensitive specimen type for detection of STIs than vaginal swab samples [47, 48]. This may be related to presence of inhibitors, or because of small proportion of the sample tested (in this case, 100 µL from several millilitres of FVU was tested). Second, the open-platform diagnostic test used in this study has not been as widely used as some of the other proprietary STI diagnostic platforms. However, the limited amount of performance data suggest good agreement with other platforms [17, 49] and we feel the flexible, transparent nature of open-platform multiplex PCR tests makes them the only viable option for this type of small scale, opportunistic study in resource-limited settings [27, 50]. Third, the participant sampling technique was not random, so we cannot assume the data are representative of the local or national Gambian populations. Although the samples were taken before any parent trial intervention, the trial was examining an HPV vaccine and recruitment relied on voluntary self-presentation to the study, both of which may have led to recruitment bias. Also, pregnant women and women who were known to be HIV positive, who may be at increased risk of STIs, were excluded. The influence of these factors should be considered when interpreting the results. Finally, as a result of the nature of the parent trial from which these specimens were taken, men were not included in our sampling frame.

Several unanswered questions remain. First, larger, randomly sampled surveys and longitudinal prevalence monitoring are needed to expand the map of STI prevalence in The Gambia. Compared to the general population, high-risk populations such as female sex workers often bear a disproportionately high proportion of STIs. Inclusion of these groups in future surveillance efforts is important. Men should also be included. Second, several key infections of the reproductive tract (for example, HIV, Herpes Simplex Virus, bacterial vaginosis, *Candida albicans*) were not tested for in this study. More data will be needed on their prevalence to ensure appropriate service provision. Third, prospectively designed STI surveys have increased scope to examine symptoms and behaviours related to STIs. Learning more about the epidemiology of these infections will be important in planning effective public health interventions. Finally, STIs, including *T. vaginalis* which was the most common in this study, are often asymptomatic, [33, 51] so proactive screening of asymptomatic men and women should be core to monitoring strategies. Given the documented limitations of urine testing, supplementing urine testing with swab sample testing in subsequent studies would be valuable. Several studies have demonstrated that self-collected vaginal swabs are acceptable and feasible [52, 53].

Our data, combined with those from elsewhere, will contribute to baseline STI prevalence estimates in The Gambia, laying a foundation for more systematic monitoring of the key challenges facing STI prevention, such as characterisation of antimicrobial susceptibility profiles among pathogen isolates and determining whether prevalence is changing over time. There is growing concern about STIs at the global level; the increasing evidence base in The Gambia should provide leverage for policy makers to ensure STIs are appropriately prioritised.

## Electronic supplementary material

Below is the link to the electronic supplementary material.

Supplementary Material 1

## Data Availability

Data available on request from corresponding author.
