# Supplementary material for: Prevalence of five treatable sexually transmitted infections among women in Lower River region of The Gambia
Source: BMC Infect Dis. 2023 Jul 13;23:471. doi: 10.1186/s12879-023-08399-2 (PMC10347728; doi:10.1186/s12879-023-08399-2)
Supplement: Supplementary file 1 — Supplementary Material 1 [file 12879_2023_8399_MOESM1_ESM.docx]

**Prevalence of five curable sexually transmitted infections among women in Lower River Region of The Gambia.**

Robert Butcher^1*^, Sheikh Jarju^2^, Dolapo Obayemi^2^, Adedapo Bashorun^2^, Hristina Vasileva^1^, Hannah Bransbury-Hare^1^, Orighomisan Agboghoroma^2^, Lamin Drammeh^2^, Martin Holland^1^, Emma Harding-Esch^1^, Ed Clarke ^1,2^.

1. Clinical Research Department, London School of Hygiene & Tropical Medicine.
2. MRC Unit The Gambia at London School of Hygiene & Tropical Medicine.

* Corresponding author. [Robert.butcher@lshtm.ac.uk](mailto:Robert.butcher@lshtm.ac.uk)

**Supplementary table 1.** Univariable and multivariable analysis of the association between having any sexually transmitted infection and demographic and behavioural characteristics of 420 Gambian women.

| **Variable** | **Level** | **N** | **n (%)** | **Univariable analysis** | |
| --- | --- | --- | --- | --- | --- |
|  |  |  |  | **Odds ratio** | **p** |
| Age group | <25 years | 194 | 16 (8) | Reference | - |
|  | ≥25 years | 226 | 25 (11) | 1.3 (0.7−2.7) | 0.334 |
| Ethnicity | Mandinka | 362 | 35 (10) | Reference | - |
|  | Other | 58 | 6 (10) | 1.1 (0.4−2.5) | 0.872 |
| Highest education level | Grade 7 and over | 213 | 19 (9) | Reference | - |
|  | Up to grade 6 | 207 | 22 (11) | 1.2 (0.6−2.3) | 0.556 |
| Past or current contraceptive usage | Yes | 127 | 17 (13) | Reference | - |
|  | No | 293 | 24 (8) | 0.6 (0.3−1.1) | 0.103 |
| Married | Yes | 248 | 24 (10) | Reference | - |
|  | No | 172 | 17 (10) | 1.0 (0.5−2.0) | 0.944 |
| Primary cooking fuel | Closed | 76 | 5 (7) | Reference | - |
|  | Open | 344 | 36 (10) | 1.7 (0.7−5.0) | 0.306 |
| Private water source | Yes | 246 | 26 (11) | Reference | - |
|  | No | 174 | 15 (9) | 0.8 (0.4−1.5) | 0.508 |
| Latrine | Improved* | 261 | 26 (10) | Reference | - |
|  | Open | 159 | 15 (10) | 0.9 (0.5−1.8) | 0.860 |
| Participant occupation | Student | 137 | 8 (6) | Reference | - |
|  | Housewife | 121 | 13 (11) | 1.9 (0.8−5.1) | 0.156 |
|  | Other work | 162 | 20 (12) | 2.3 (1.0−5.6) | 0.060 |
| * Private or shared | | | | | |
